# Trends in Diagnosis of HIV Infection, Linkage to Medical Care, and Viral Suppression Among Men Who Have Sex with Men, by Race/Ethnicity and Age — 33 Jurisdictions, United States, 2014–2018

**DOI:** 10.15585/mmwr.mm6938a1

**Published:** 2020-09-25

**Authors:** William L. Jeffries, André F. Dailey, Chan Jin, Jarvis W. Carter, Lamont Scales

**Affiliations:** ^1^Division of HIV/AIDS Prevention, National Center for HIV/AIDS, Viral Hepatitis, STD, and TB Prevention, CDC; ^2^ICF International, Inc., Atlanta, Georgia

During 2018, gay, bisexual, and other men who have sex with men (MSM) accounted for 69.4% of all diagnoses of human immunodeficiency virus (HIV) infection in the United States (*1*). Moreover, in all 42 jurisdictions with complete laboratory reporting of CD4 and viral load results,* percentages of MSM linked to care within 1 month (80.8%) and virally suppressed (viral load <200 copies of HIV RNA/mL or interpreted as undetected) within 6 months (68.3%) of diagnosis were below target during 2018 (*2*). African American/Black (Black), Hispanic/Latino (Hispanic), and younger MSM disproportionately experience HIV diagnosis, not being linked to care, and not being virally suppressed. To characterize trends in these outcomes, CDC analyzed National HIV Surveillance System^†^ data from 2014 to 2018. The number of diagnoses of HIV infection among all MSM decreased 2.3% per year (95% confidence interval [CI] = 1.9–2.8). However, diagnoses did not significantly change among either Hispanic MSM or any MSM aged 13–19 years; increased 2.2% (95% CI = 1.0–3.4) and 2.0% (95% CI = 0.6–3.3) per year among Black and Hispanic MSM aged 25–34 years, respectively; and were highest in absolute count among Black MSM. Annual percentages of linkage to care within 1 month and viral suppression within 6 months of diagnosis among all MSM increased (2.9% [95% CI = 2.4–3.5] and 6.8% [95% CI = 6.2–7.4] per year, respectively). These findings, albeit promising, warrant intensified prevention efforts for Black, Hispanic, and younger MSM.

CDC used data reported to the National HIV Surveillance System by December 2019 to identify cases of HIV infection that met CDC’s HIV infection case definition among MSM, including MSM aged ≥13 years who inject drugs (*3*). Multiple imputation was used to adjust for unknown or missing transmission category (15.6% of cases) (*4*). At the time of diagnosis, all MSM resided in one of 33 jurisdictions^§^ with complete laboratory reporting for each year during 2014–2018. Linkage-to-care analyses included MSM with HIV infection diagnosed during the calendar year when the diagnosis was first made. Linkage to care was defined as one or more CD4 or viral load tests performed within 1 month of diagnosis. Viral suppression within 6 months of diagnosis was measured for MSM whose infection was diagnosed during the outcome year and who resided in any of the 33 jurisdictions at the time of diagnosis of HIV infection. Viral suppression was defined as a viral load result of <200 copies/mL or a viral load test interpretation value of undetected.

Results are presented by race/ethnicity (Black, Hispanic, other, and White) and age group (13–19, 20–24, 25–34, 35–44, 45–54, and ≥55 years). The estimated annual percentage change (EAPC) was calculated for each MSM group. Because of unknown population denominators, case counts were used to analyze diagnoses by transmission category; the EAPCs in case counts were calculated by using a Poisson distribution. EAPCs indicate the per-year change, on average, in the number of diagnoses, percentage linked to care, or percentage virally suppressed. EAPC p-values <0.05 indicated statistically significant trends, whereas p-values ≥0.05 indicated no significant change. Analyses were conducted using SAS (version 9.4; SAS Institute).

During 2014–2018, the number of diagnoses of HIV infection among all MSM decreased 2.3% (95% CI = 1.9–2.8) per year (from 19,789 to 18,034), on average (Table 1). Among Black MSM, diagnoses decreased 1.3% per year overall and 6.0% and 5.6% among those aged 20–24 and 45–54 years, respectively. Diagnoses did not significantly change among Black MSM aged 13–19, 35–44, and ≥55 years, but increased 2.2% annually among those aged 25–34 years. Among Hispanic MSM, diagnoses did not significantly change overall or among those aged 13–19, 35–44, 45–54, and ≥55 years. Diagnoses decreased 3.7% per year among Hispanic MSM aged 20–24 years but increased 2.0% among those aged 25–34 years. Among White MSM, diagnoses decreased 4.8% per year overall and 5.6%, 2.1%, 7.8%, and 9.3% among those aged 20–24, 25–34, 35–44, and 45–54 years, respectively. Diagnoses did not significantly change among White MSM aged 13–19 or ≥55 years.

**TABLE 1:** Diagnoses of human immunodeficiency virus (HIV) infection and linkage to medical care within 1 month of diagnosis among men who have sex with men,* by race/ethnicity and age — 33 jurisdictions,^†^ United States, 2014–2018^§^

Race/Ethnicity	Diagnoses, no.					EAPC^¶^ (95% CI)	Linkage to medical care, no. (%)					EAPC^¶^ (95% CI)
	2014	2015	2016	2017	2018	2014–2018	2014	2015	2016	2017	2018	2014–2018
**African American/Black**												
**Age at diagnosis (yrs)**												
13–19	574	613	593	600	584	0.1 (−2.4 to 2.7)	335 (58.4)	376 (61.3)	388 (65.4)	417 (69.5)	399 (68.3)	4.4 (1.2 to 7.8)
20–24	2,262	2,163	2,085	1,861	1,784	−6.0 (−7.3 to −4.7)	1,243 (55.0)	1,279 (59.1)	1,326 (63.6)	1,216 (65.3)	1,232 (69.1)	5.7 (3.9 to 7.6)
25–34	2,627	2,731	2,853	2,872	2,860	2.2 (1.0 to 3.4)	1,618 (61.6)	1,685 (61.7)	1,879 (65.8)	1,945 (67.7)	2,024 (70.8)	3.8 (2.3 to 5.3)
35–44	925	912	908	911	933	0.2 (−1.9 to 2.2)	609 (65.9)	593 (65.0)	605 (66.6)	641 (70.4)	662 (71.0)	2.3 (−0.2 to 4.9)
45–54	624	618	583	520	509	−5.6 (−8.0 to −3.1)	423 (67.8)	410 (66.4)	387 (66.3)	356 (68.5)	350 (68.8)	0.6 (−2.6 to 3.8)
≥55	317	278	290	310	297	−0.3 (−3.8 to 3.4)	222 (70.1)	184 (66.2)	198 (68.2)	207 (66.8)	214 (72.1)	0.6 (−3.6 to 5.0)
**Subtotal**	**7,328**	**7,314**	**7,312**	**7,074**	**6,967**	**−1.3 (−2.0 to −0.6)**	**4,450 (60.7)**	**4,525 (61.9)**	**4,781 (65.4)**	**4,783 (67.6)**	**4,881 (70.1)**	**3.8 (2.9 to 4.8)**
Hispanic/Latino**												
**Age at diagnosis (yrs)**												
13–19	222	234	228	242	222	0.4 (−3.6 to 4.6)	131 (59.1)	157 (67.2)	154 (67.5)	156 (64.2)	163 (73.4)	3.8 (−1.3 to 9.3)
20–24	1,130	1,170	1,108	1,027	995	−3.7 (−5.5 to −1.9)	719 (63.6)	775 (66.2)	763 (68.9)	706 (68.8)	736 (74.0)	3.4 (1.1 to 5.8)
25–34	2,071	2,100	2,264	2,226	2,221	2.0 (0.6 to 3.3)	1,391 (67.2)	1,433 (68.3)	1,659 (73.2)	1,647 (74.0)	1,679 (75.6)	3.2 (1.6 to 4.8)
35–44	1,158	1,125	1,106	1,113	1,071	−1.6 (−3.5 to 0.2)	806 (69.6)	806 (71.7)	807 (73.0)	837 (75.2)	869 (81.1)	3.6 (1.4 to 5.9)
45–54	594	648	569	597	590	−1.0 (−3.5 to 1.5)	416 (70.0)	463 (71.4)	437 (76.8)	455 (76.2)	464 (78.7)	3.0 (0.1 to 6.1)
≥55	191	205	199	213	231	4.4 (0.0 to 9.0)	152 (79.9)	153 (74.4)	151 (75.9)	166 (78.0)	177 (76.6)	−0.3 (−5.1 to 4.7)
**Subtotal**	**5,366**	**5,482**	**5,473**	**5,417**	**5,331**	**−0.2 (−1.1 to 0.6)**	**3,616 (67.4)**	**3,787 (69.1)**	**3,970 (72.5)**	**3,967 (73.2)**	**4,089 (76.7)**	**3.2 (2.2 to 4.3)**
**Other race/ethnicity**												
**Age at diagnosis (yrs)**												
13–19	67	75	65	63	53	−6.0 (−13.0 to 1.5)	39 (58.0)	44 (58.6)	46 (70.9)	48 (76.3)	43 (81.0)	9.9 (−0.2 to 20.9)
20–24	332	337	305	286	215	−9.2 (−12.4 to −5.8)	203 (61.1)	237 (70.4)	215 (70.4)	209 (73.0)	170 (79.1)	5.6 (1.0 to 10.4)
25–34	568	613	605	528	499	−3.9 (−6.4 to −1.3)	408 (71.9)	457 (74.6)	444 (73.3)	405 (76.8)	376 (75.2)	1.2 (−1.9 to 4.4)
35–44	313	269	278	259	216	−7.4 (−10.8 to −3.8)	233 (74.4)	202 (75.1)	218 (78.3)	200 (77.3)	175 (80.7)	1.9 (−2.4 to 6.5)
45–54	199	181	157	179	122	−8.9 (−13.2 to −4.4)	150 (75.3)	138 (76.4)	122 (78.0)	142 (79.4)	106 (86.4)	3.0 (−2.5 to 8.8)
≥55	58	70	87	60	65	0.5 (−6.8 to 8.3)	37 (64.3)	52 (74.4)	65 (74.3)	45 (75.9)	54 (83.9)	5.5 (−3.7 to 15.6)
**Subtotal**	**1,537**	**1,544**	**1,495**	**1,375**	**1,170**	**−6.1 (−7.7 to −4.6)**	**1,070 (69.6)**	**1,130 (73.2)**	**1,108 (74.1)**	**1,050 (76.3)**	**923 (78.9)**	**3.0 (1.0 to 5.0)**
**White**												
**Age at diagnosis (yrs)**												
13–19	105	97	121	121	115	4.0 (−1.9 to 10.3)	56 (53.4)	64 (66.0)	79 (65.2)	77 (63.7)	78 (68.1)	4.4 (−3.1 to 12.5)
20–24	753	671	637	675	560	−5.6 (−7.9 to −3.3)	461 (61.2)	451 (67.3)	417 (65.4)	503 (74.6)	405 (72.2)	4.6 (1.5 to 7.7)
25–34	1,700	1,740	1,617	1,586	1,605	−2.1 (−3.6 to −0.6)	1,179 (69.3)	1,261 (72.4)	1,178 (72.8)	1,154 (72.8)	1,246 (77.6)	2.4 (0.5 to 4.2)
35–44	1,213	1,072	943	905	888	−7.8 (−9.6 to −6.0)	912 (75.2)	809 (75.4)	702 (74.4)	702 (77.5)	694 (78.2)	1.0 (−1.2 to 3.3)
45–54	1,178	1,082	1,034	888	791	−9.3 (−11.1 to −7.5)	904 (76.7)	832 (76.9)	824 (79.7)	689 (77.6)	628 (79.4)	0.8 (−1.5 to 3.1)
≥55	610	585	621	573	607	−0.3 (−2.8 to 2.3)	450 (73.9)	450 (77.0)	479 (77.1)	438 (76.4)	470 (77.4)	0.9 (−2.0 to 3.8)
**Subtotal**	**5,559**	**5,247**	**4,973**	**4,748**	**4,566**	**−4.8 (−5.7 to −4.0)**	**3,961 (71.3)**	**3,867 (73.7)**	**3,678 (74.0)**	**3,564 (75.1)**	**3,521 (77.1)**	**1.8 (0.7 to 2.8)**
**Total**	**19,789**	**19,586**	**19,254**	**18,614**	**18,034**	**−2.3 (−2.8 to −1.9)**	**13,097 (66.2)**	**13,308 (67.9)**	**13,538 (70.3)**	**13,362 (71.8)**	**13,414 (74.4)**	**2.9 (2.4 to 3.5)**

**Abbreviations:** CI = confidence interval; EAPC = estimated annual percentage change.

* Men who have sex with men were persons whose sex at birth was male and whose transmission category was either male-to-male sexual contact or male-to-male sexual contact and injection drug use.

^†^ Data are based on residence at time of diagnosis of HIV infection. The 33 jurisdictions were Alabama, Alaska, California, District of Columbia, Georgia, Hawaii, Illinois, Indiana, Iowa, Louisiana, Maine, Maryland, Massachusetts, Michigan, Minnesota, Mississippi, Missouri, Nebraska, New Hampshire, New Mexico, New York, North Dakota, Oregon, South Carolina, South Dakota, Tennessee, Texas, Utah, Virginia, Washington, West Virginia, Wisconsin, and Wyoming.

^§^ Data have been statistically adjusted by using multiple imputation to account for unknown or missing transmission category; therefore, values might not sum to column subtotals and total.

^¶^ EAPCs indicate the per-year change, on average, in the number of diagnoses of HIV infection or percentage linked to medical care. EAPC p-values <0.05 indicated statistically significant trends, whereas EAPC p-values ≥0.05 indicated no significant trend.

** Hispanics/Latinos might be of any race.

The percentage of all MSM who were linked to care within 1 month of diagnosis increased 2.9% per year, on average, from 2014 (66.2%) to 2018 (74.4%). Among Black MSM, the percentage linked to care increased 3.8% per year overall, and it increased among those aged 13–19, 20–24, and 25–34 years. It did not significantly change among those aged 35–44, 45–54, and ≥55 years. Among Hispanic MSM, the percentage linked to care increased 3.2% per year overall, and it increased among those aged 20–24, 25–34, 35–44, and 45–54 years. However, the percentage linked to care did not significantly change among those aged 13–19 and ≥55 years. Among White MSM, the percentage linked to care increased 1.8% per year overall, and it increased among those aged 20–24 and 25–34 years but did not significantly change among all other age groups.

The percentage of all MSM who achieved viral suppression within 6 months of diagnosis increased 6.8% per year, on average, from 2014 (51.1%) to 2018 (67.2%) (Table 2). Among Black MSM, the percentage who achieved viral suppression increased 9.4% per year overall, and it increased among those aged 13–19, 20–24, 25–34, 35–44, and 45–54 years. The percentage virally suppressed did not significantly change among Black MSM aged ≥55 years. Among Hispanic MSM, the percentage who were virally suppressed increased 6.8% per year overall, and it increased among those aged 20–24, 25–34, 35–44, and 45–54 years; it did not significantly change among those aged 13–19 or ≥55 years. The percentage of White MSM who achieved viral suppression increased 4.4% per year overall, and it increased among those aged 13–19, 20–24, 25–34, 35–44, and 45–54 years; it did not significantly change among those aged ≥55 years.

**TABLE 2:** Viral suppression within 6 months of diagnosis among men who have sex with men,* by race/ethnicity and age — 33 jurisdictions,^†^ United States, 2014–2018^§^

Race/Ethnicity	No. (%)					EAPC^¶^ (95% CI)
	2014	2015	2016	2017	2018	2014–2018
**African American/Black**						
**Age at diagnosis (yrs)**						
13–19	220 (38.4)	277 (45.2)	304 (51.3)	365 (60.9)	372 (63.7)	13.8 (9.7 to 17.9)
20–24	865 (38.2)	966 (44.7)	1,064 (51.0)	985 (52.9)	1,085 (60.8)	11.5 (9.3 to 13.8)
25–34	1,134 (43.2)	1,302 (47.7)	1,495 (52.4)	1,637 (57.0)	1,764 (61.7)	9.3 (7.5 to 11.1)
35–44	445 (48.1)	468 (51.4)	494 (54.4)	533 (58.6)	590 (63.2)	7.0 (4.1 to 10.0)
45–54	313 (50.1)	322 (52.2)	317 (54.3)	309 (59.4)	303 (59.5)	4.8 (1.2 to 8.6)
≥55	153 (48.2)	150 (53.9)	151 (52.1)	153 (49.3)	180 (60.4)	3.8 (−1.1 to 9.1)
**Subtotal**	**3,130 (42.7)**	**3,485 (47.6)**	**3,826 (52.3)**	**3,982 (56.3)**	**4,294 (61.6)**	**9.4 (8.3 to 10.5)**
Hispanic/Latino**						
**Age at diagnosis (yrs)**						
13–19	112 (50.7)	128 (54.5)	127 (55.7)	135 (55.5)	145 (65.3)	5.4 (−0.2 to 11.4)
20–24	533 (47.2)	621 (53.1)	639 (57.7)	641 (62.4)	642 (64.5)	8.1 (5.4 to 10.9)
25–34	1,088 (52.5)	1,200 (57.2)	1,403 (62.0)	1,439 (64.7)	1,592 (71.7)	7.7 (5.9 to 9.6)
35–44	647 (55.9)	687 (61.1)	663 (60.0)	727 (65.3)	775 (72.3)	6.1 (3.6 to 8.6)
45–54	338 (56.9)	395 (60.9)	360 (63.2)	384 (64.4)	403 (68.4)	4.3 (1.0 to 7.7)
≥55	111 (58.0)	121 (58.8)	139 (69.8)	131 (61.6)	151 (65.4)	2.8 (−2.6 to 8.5)
**Subtotal**	**2,829 (52.7)**	**3,152 (57.5)**	**3,330 (60.9)**	**3,456 (63.8)**	**3,708 (69.6)**	**6.8 (5.6 to 8.0)**
**Other race/ethnicity**						
**Age at diagnosis (yrs)**						
13–19	31 (46.0)	35 (46.5)	35 (53.9)	43 (68.2)	37 (69.6)	13.2 (1.8 to 25.8)
20–24	146 (44.0)	199 (59.2)	176 (57.7)	188 (65.7)	166 (77.2)	12.7 (7.4 to 18.3)
25–34	322 (56.7)	393 (64.1)	396 (65.4)	371 (70.3)	350 (70.1)	5.2 (1.8 to 8.8)
35–44	202 (64.5)	166 (61.8)	203 (73.0)	192 (74.0)	156 (72.2)	4.2 (−0.5 to 9.1)
45–54	119 (59.5)	121 (66.7)	106 (68.0)	118 (65.7)	95 (77.9)	5.1 (−0.9 to 11.6)
≥55	32 (55.2)	43 (62.2)	49 (56.1)	37 (62.6)	46 (70.5)	5.2 (−4.9 to 16.4)
**Subtotal**	**851 (55.4)**	**957 (62.0)**	**964 (64.5)**	**949 (69.0)**	**850 (72.6)**	**6.7 (4.5 to 9.0)**
**White**						
**Age at diagnosis (yrs)**						
13–19	50 (47.7)	56 (57.9)	69 (57.0)	79 (65.3)	81 (70.7)	9.5 (1.3 to 18.3)
20–24	391 (51.9)	403 (60.0)	373 (58.6)	450 (66.7)	382 (68.2)	6.8 (3.5 to 10.2)
25–34	964 (56.7)	1,098 (63.1)	1,035 (64.0)	1,058 (66.7)	1,147 (71.4)	5.3 (3.3 to 7.3)
35–44	748 (61.7)	693 (64.6)	632 (67.0)	620 (68.5)	648 (73.0)	4.0 (1.6 to 6.5)
45–54	754 (64.1)	708 (65.4)	725 (70.2)	614 (69.2)	567 (71.6)	2.9 (0.4 to 5.4)
≥55	390 (64.0)	378 (64.6)	404 (65.0)	397 (69.3)	433 (71.4)	2.9 (−0.2 to 6.2)
**Subtotal**	3,297 (59.3)	3,335 (63.6)	3,239 (65.1)	3,219 (67.8)	3,258 (71.4)	4.4 (3.3 to 5.6)
**Total**	**10,107 (51.1)**	**10,928 (55.8)**	**11,359 (59.0)**	**11,607 (62.4)**	**12,110 (67.2)**	**6.8 (6.2 to 7.4)**

**Abbreviations:** CI = confidence interval; EAPC = estimated annual percentage change; HIV = human immunodeficiency virus.

* Men who have sex with men were persons whose sex at birth was male and whose transmission category was either male-to-male sexual contact or male-to-male sexual contact and injection drug use.

^†^ Data are based on residence at time of diagnosis of HIV infection. The 33 jurisdictions were Alabama, Alaska, California, District of Columbia, Georgia, Hawaii, Illinois, Indiana, Iowa, Louisiana, Maine, Maryland, Massachusetts, Michigan, Minnesota, Mississippi, Missouri, Nebraska, New Hampshire, New Mexico, New York, North Dakota, Oregon, South Carolina, South Dakota, Tennessee, Texas, Utah, Virginia, Washington, West Virginia, Wisconsin, and Wyoming.

^§^ Data have been statistically adjusted by using multiple imputation to account for unknown or missing transmission category; therefore, values might not sum to column subtotals and total.

^¶^ EAPCs indicate the per-year change, on average, in the percentage virally suppressed. EAPC p-values <0.05 indicated statistically significant trends, whereas EAPC p-values ≥0.05 indicated no significant trend.

** Hispanics/Latinos can be of any race.

## Discussion

Annual diagnoses of HIV infection among MSM in the 33 analyzed jurisdictions decreased during 2014–2018. However, the rate of annual decrease among Black MSM (1.3%) was less than that among White MSM (4.8%), diagnoses did not significantly change among Hispanic MSM or any MSM aged 13–19 years, and diagnoses increased among Black and Hispanic MSM aged 25–34 years. In addition, more diagnoses occurred overall among Black MSM than among other racial/ethnic MSM groups. CDC recently reported that racial/ethnic disparities in estimated rates of diagnosis of HIV infection among MSM increased during 2010–2015, and Black MSM had an HIV diagnosis rate that was 9.3 times that of White MSM in 2015 (*5*). These data warrant intensified prevention efforts for Black and Hispanic MSM, especially those aged 25–34 years, and all MSM aged 13–19 years.

Increased linkage to care promotes viral suppression, which effectively prevents HIV transmission. During 2014–2018, linkage to care within 1 month and viral suppression within 6 months of diagnosis increased (2.9% and 6.8% per year, respectively). Increases were highest among Black and Hispanic MSM. However, among all MSM included in the 2018 analysis, only 67.2% achieved viral suppression within 6 months of diagnosis. Moreover, during 2018, proportionally fewer Black MSM were linked to care and achieved viral suppression than did other racial/ethnic MSM groups. Limited health care access, housing instability, poverty, and systemic racism commonly impede linkage to care and viral suppression (*6*,*7*). Addressing these factors might improve outcomes.

The findings in this report are subject to at least two limitations. First, only 33 of the 51 U.S. jurisdictions had complete laboratory reporting of CD4 and viral load results during 2014–2018. Therefore, data do not represent all diagnoses of HIV infection among MSM during 2014–2018. Second, using EAPCs with p-values <0.05 to identify trends might result in clinically meaningful temporal changes being deemed as having no significant change.

Providing antiretroviral therapy for both HIV preexposure prophylaxis and treatment can prevent HIV infection and, subsequently, the need for linkage to care and viral suppression among MSM (*8*,*9*). However, during 2017, Black and Hispanic MSM who had discussed preexposure prophylaxis with a medical provider were less likely than were White MSM to receive prescriptions for preexposure prophylaxis in 23 jurisdictions (*8*). Providers’ implicit racial biases toward Blacks and Hispanics often promote treatment nonadherence (*10*), which inhibits viral suppression (*9*). Therefore, interventions might need to address systemic racism and concomitant racial biases within health care systems (*7*). CDC encourages use of interventions that address social determinants of health^¶^ that underlie the high risk for HIV infection among MSM of all races/ethnicities and ages. Such interventions might help prevent HIV infection and eliminate racial/ethnic disparities in HIV infection among MSM.

**Summary**

**What is already known about this topic?**

Men who have sex with men (MSM) account for two thirds of annual diagnoses of human immunodeficiency virus (HIV) infection. Increased linkage to care and viral suppression among MSM with HIV infection can prevent transmission.

**What is added by this report?**

During 2014–2018, diagnoses of HIV infection among MSM in 33 jurisdictions decreased 2.3% per year overall, but Black, Hispanic/Latino, and younger (aged 13–19 years) MSM experienced a small or no decrease. Linkage to care within 1 month and viral suppression within 6 months of diagnosis increased overall (2.9% and 6.8% per year, respectively) and among all racial/ethnic groups.

**What are the implications for public health practice?**

Intensified prevention efforts for Black, Hispanic/Latino, and younger MSM are needed.

## Footnotes

* CDC established three criteria for complete laboratory reporting: 1) the jurisdiction’s laws/regulations required reporting of all CD4 and viral load results to the state or local health department; 2) laboratories that perform HIV-related testing for the areas must have reported a minimum of 95% of HIV-related test results to the state or local health department; and 3) by December 31, 2019, the jurisdiction had reported to CDC at least 95% of all CD4 and viral load results received during January 2017–September 2019. Additional information is available at https://www.cdc.gov/hiv/pdf/library/reports/surveillance/cdc-hiv-surveillance-supplemental-report-vol-25-2.pdf.

† The National HIV Surveillance System is the primary source for monitoring HIV trends in the United States. Through the system, CDC funds and assists state and local health departments collecting data on cases of HIV infection. Health departments provide deidentified data to CDC.

§ Alabama, Alaska, California, District of Columbia, Georgia, Hawaii, Illinois, Indiana, Iowa, Louisiana, Maine, Maryland, Massachusetts, Michigan, Minnesota, Mississippi, Missouri, Nebraska, New Hampshire, New Mexico, New York, North Dakota, Oregon, South Carolina, South Dakota, Tennessee, Texas, Utah, Virginia, Washington, West Virginia, Wisconsin, and Wyoming.

¶ https://www.cdc.gov/socialdeterminants/docs/sdh-white-paper-2010.pdf.
